# Possible effect of biotechnology on plant gene pools in Turkey

**DOI:** 10.1080/13102818.2014.981368

**Published:** 2014-11-26

**Authors:** Aynur Demir

**Affiliations:** ^a^ Department of Environmental Protection Technologies, Technical Sciences Vocational School, Aksaray University, Aksaray, Turkey

**Keywords:** plant gene pools, biotechnology, agricultural biological diversity, gene flow, Turkey

## Abstract

The recent rapid developments in biotechnology have made great contributions to the study of plant gene pools. The application of *in vitro* methods in freeze storage and DNA protection techniques in fast production studies has made major advances. From that aspect, biotechnology is an indispensable means for the protection of plant gene pools, which includes the insurance of sustainable agriculture and development of species. Besides all the positive developments, one of the primary risks posed by the uncontrolled spreading of genetically modified organisms is the possibility for other non-target organisms to be negatively affected. Genes of plant origin should be given priority in this type of studies by taking into consideration such negative effects that may result in disruption of ecological balance and damage to plant genetic pools. Turkey, due to its ecological conditions and history, has a very important position in terms of plant gene pools. This richness ought to be protected without corrupting its natural quality and natural evolution process in order to provide the sources of species that will be required for future sustainable agricultural applications. Thus, attention should be paid to the use of biotechnological methods, which play an important role especially in the protection and use of local and original plant gene pools.

## Introduction

Plant biological diversity is an important factor particularly in such fields as agriculture, medicine, pharmacy and environment and is also an indicator of development potential. In view of the depletion of non-renewable resources, plant biological diversity is crucial for sustaining human activity in the future. In this situation, rational and sustainable use of plant gene pools is needed, putting emphasis on the need to take precautions for their protection.

Biotechnology is defined as ‘any technological application that uses biological systems, living organisms, or derivatives thereof, to make or modify products or processes for specific use’ in the Convention on Biological Diversity,[1] which has been developed to ensure fair and equitable sharing of the benefits arising from the use of biological diversity elements, which constitutes the main guarantee of sustainable development, and to which Turkey is a party. As highlighted here, biotechnology refers to any kind of technological application adopted in the process of changing the genetic structure of living organisms to produce desired genotypes. One of these applications is plant biotechnology. Plant biotechnology, in its own right, is described as the biotechnological methods applied in order to contribute to the production and improvement of plant species or to offer solutions for the problems that cannot be solved or overcome via traditional methods and to achieving better quality and more economical plant production.[2,3] Thus, plant gene pools are used as the main material in many areas of applied biotechnology. Plant biodiversity has been recognized as a source of wealth for the future. In this context, biological diversity is an indicator of a healthy environment and at the same time it reflects the potential of ecosystems’ life-support processes, which is necessary for the welfare of human beings. These sustainable processes provide the systems needed to support living communities and thus a critical interdependence is created. This dependence, in a sense, is the phenomenon underlying the sustainable development approach. Biotechnology, on the other hand, is, in a way, insurance of the use of biological gene pools in line with the principles of sustainable development.

Particularly, when the changing climate conditions are taken into consideration, biotechnology applications further raise the expectations for sustainable use of plant gene pools as well. While these technological methods, on the one hand, offer both economical and ecological facilitations in the *ex situ* protection and enrichment of gene pools and production of new products, they may, on the other hand, result in extinction of local or wild species and undesired pollination and thus, negatively affect natural evolution. These risks pose a potential problem particularly for countries rich in plant genetic diversity, such as Turkey. This forms the focal point of discussions on biotechnological applications and places the so-called ‘precautionary principle’ in applications high on the agenda. While the countries with rich biodiversity regard biotechnology as an opportunity and target utilizing the possibilities it offers, they are concerned about the potential risks it poses and follow the precautionary path in applications. In line with this, the present review discusses the potential effects of modern biotechnological applications, within the framework of the plant genetic diversity in Turkey, on the protection and use of plant diversity, by addressing the positive and negative aspects of these applications. The importance of the plant gene pools of Turkey in biotechnology is emphasized via evaluations made on the basis of sample species.

The study is based on a review of published reports, and a descriptive approach is adopted in the evaluation of existing data. The evaluation is generally shaped on research works, which focus on the use of biotechnology in the utilization of plant gene sources in Turkey and the development of agricultural products, and the situation is interpreted analytically.

## Plant gene pools and biodiversity in Turkey

Located within the temperate zone, Turkey attracts attention with its outstanding plant diversity characteristics different from many of its neighbours. The number of plant species prevailing in Turkey is close to the sum of the number of plant species prevailing on the whole European continent. After the discoveries made in recent years, Turkey has been revealed to have around 12,000 plant taxa (at species, subspecies or variety level), 3000 of them being endemic.[4] With its 34.4% endemism ratio, Turkey is also one of the richest countries in Europe in terms of endemic species diversity.[5–7]

This characteristic results from the diverse geography of Turkey. Many geographical factors such as short-distance changes in climate conditions, diversity caused by geographical location, vegetation and diversity in soil types have led to differentiation of plant formations and diversification of species. Surrounded by sea on three sides, Turkey has mountainous regions rising immediately behind the north and south shores and elevation differences which become more noticeable moving from west to east, which have resulted in diversifications in plant communities. In addition to these diverse geographical factors, Turkey is located at the intersection point of two important Vavilov centres of diversity, i.e. the Mediterranean and the Middle East, which brings about high plant and genetic diversity. Moreover, Anatolia is located on historical migration routes and has hosted many civilizations throughout its history. These two factors have played a significant role in the increase in plant diversity and richness as well as in the diversification of gene pools.

In addition, the diversity and richness of plant genetic sources in Turkey is closely related to the fact that Anatolia falls within three different phytogeographical regions, i.e. the genetic centres of the Euro–Siberian, the Mediterranean and Irano–Turanian flora. The most common species of the Euro–Siberian flora include fir (*Abies* sp.), spruce (*Picea oriantalis*), pine (*Pinus sylvestris*), yew (*Taxsus baccata*) and rhododendrons (*Rhodendron sp*); of the Mediterranean flora, Taurus fir (*Abies cilicica*), Lebanon cedar (*Cedrus libani*), black pine (*Pinus nigra*), Calabrian pine (*Pinus burtia*), cypress (*Cupressus sempervirens)* and scrub communities; and of the Irano–Turanian flora, milkvetch (*Astragalus*), prickly thrift (*Acantholimon*) and the flora of real steppe lands such as the Lake Tuz (Salt Lake) region or Igdir plain.[8]

Turkey's location has also played an important role in the introduction of cereals and garden plants. There are five different ‘micro-genetic centres’ in Turkey, where more than 100 species show a wide distribution (Table 1).[9] There are 256 cereal species developed on the basis of local and import breeds and recorded in the last three decades; 95 of these 256 species are wheat, 91 are corn, 22 are barley, 19 are rice, 16 are sorghum, 11 are oat and 2 are rye species.[10]

**Table 1.** Micro-genetic centres in Turkey and common species.

Micro-genetic centres	Species
Thrace–Aegean	Wheat (bread, durum, oriental, club, einkorn, spelt), grits, sweet melon, lentil, chickpea, common vetch, lupine, clover.
Southeastern Anatolia	Einkorn, emmer, *Ae. speltoides*, vegetable marrow, watermelon, sweet melon, cucumber, grape fern, common bean, lentil, chickpea, broad bean, fodder crops.
Samsun–Tokat–Amasya	Fruit genera and species, common bean, lentil, broad bean, legume fodder crops.
Kayseri and its surroundings	Apple, almond, pear, fruit species, grape fern, lentil, chickpea, trefoil, sainfoin.
Agrı and its surroundings	Apple, apricot, sour cherry, cherry, sweet melon, legume fodder crops.

The Turkish flora also includes wild relatives of the important cultured agricultural plant species and the genetic diversity related to these species. The number of local and other species of garden plants is thought to reach 200, including nearly 50 genera which are grown at present and nearly 100 species that have been commercially propagated. This diversity is clearly noticeable in fruit species as well, whose number is estimated to be around 138.[9]

Some important crop plants which are genetically and originally centred in Turkey can be listed as follows: *Triticum*, *Hordeum*, *Secale*, *Avena*, *Linum*, *Allium*, *Cicer*, *Lens*, *Pisum*, *Medicago* and *Vicia*. Wheat (*Triticum* and *Aegilops*) has 25 wild relatives in Turkey, barley (*Hordeum*) has 8, rye (*Secale*) has 5 and oat (*Avena*) has 8 ones. Turkey is also rich in wild relatives of edible legume and fodder crops: Turkey hosts 4 species of lentil (*Lens*); 10 species of cicer (*Cicer*); 104 species of white clover (*Trifolium*), 11 of them being endemic; 34 species of burclover (*Medicago*); 42 species of Sainfoins (*Onobrychis*); and 60 species of common vetch (*Vicia*), 6 of them endemic.[11] At the same time, Turkey is a micro-genetic centre of the species *Amygdalus* spp., *Cucumis melo*, *C. sativus*, *Cucurbita moshata*, *C. pepo*, *Malus spp.*, *Pistachio spp.*, *Prunus spp.*, *Pyrus spp.*, *Vitis silvestris* and *Vitis vinifera.*[12] Besides, mullein (*Verbascum*), sideritis (*Sideritis*), bachelor's button (*Centaurea*) and milkvetch (*Astragalus*) are among the plant groups with the highest endemism rates in Anatolia.[4]

## Role of biotechnology in studies on protection of plant gene pools in Turkey

Plant gene pools – an indicator of the ability of ecosystems to sustain life as required for human beings’ welfare and a healthy environment – have started to be exploited at a growing scale to meet both basic and other needs of human beings. The pressure put particularly on plant gene pools with the increase in human population has necessitated development of protection policies for sustainability of these resources and use of biotechnology in putting these policies into practice. At the same time, biotechnology has played a vital role in the protection and improvement of biological diversity to date.

Globally, two basic strategies are adopted in protection of genetic diversity: *in situ* and *ex situ* protection strategies. *In situ* protection means protection of ecosystems and natural habitats; ensuring the sustainability of populations of species living in their natural environment; protection of the cultured species in the environments where they grow and adopt new characteristics.[1] Since the natural evolution process continues during the *in situ* protection process, this protection strategy should be further elaborated and generalized. *Ex situ* protection, on the other hand, means protection of the basic elements of biological diversity outside their natural habitat.[1] *Ex situ* protection is performed in areas such as seed banks, DNA gene banks, pollen banks, *in vitro* gene banks, species protection gardens, arboretum, herbarium and botanic gardens.[13,14] Modern biotechnology plays a key role mainly in *ex situ* protection systems. In terms of plant genetic pools, the *ex situ* protection strategy employs biotechnology in embryo culture, embryo rescue, tissue culture and protection under extra cold conditions of (1) recalcitrant seed species (oak, Sweet Chestnut), for which traditional protection methods cannot be applied, (2) species difficult to breed from seeds (orchids, ornamental plants), (3) species reproducing vegetatively (fruit trees), (4) tuberous, rhizomatous and bulbous species (*Galanthus*, *Orchidaceae*, *Sternbergia*). Furthermore, modern biotechnology is also employed in applications such as DNA and pollen protection. Biotechnological methods enable (1) estimation of genetic diversity within and between the populations, (2) enlargement of the available plant genetic pools, using modern improvement techniques and (3) genetic mapping of plant species.

Thus, it is possible to define the locations to focus on the material collection studies, and owing to the knowledge of the range of diversity change of the genetic material stored in gene banks, collection accessions with similar genetic structure can be eliminated. Genetic material can be classified via molecular methods and, thus, re-collection of the same sample is prevented. For instance, tomato diversity studies using restriction fragment length polymorphism have laid the ground for introduction of new genes, 20-fold higher than the number of genes introduced by classical methods.[15,16] This strategy is quite effective in the protection, storage and commercial production of local tomato species. On the other hand, collection of DNA samples from non-living material – such as herbarium – and comparison of these samples with the existing materials serves as an important factor in genetic material identification. In this way, a lower number of materials can be stored and gene pool studies become more economical; consequently, there would be more free place for new gene sources.

The Turkish Seed Gene Bank [10], established in 2010 and ranking among the known gene centres of the world, has a sample capacity of 250,000 genes. The Turkish Seed Gene Bank is an important step of *ex situ* studies, with modern biotechnology applications. Owing to biotechnological characterization studies, duplicate entry of the same material is prevented, exact ‘finger prints’ of the genotypes are created, genetic diversity is detected either in the gene bank or under natural conditions and core collections are formed within large collections in order to represent an important part of diversity with a minimum number of samples. At present, 69,000 seed samples from a total of 2500 species are stored in this gene bank. In addition, small gene centres located in different areas have taken under protection a gene pool of 6210 fruit species samples and 2132 grape fern samples belonging to 51 species; 11,500 samples of 8 plant species; and 263 plant species collected from 1200 locations in the nature.[15,17] The aim of these gene centres is to collect, record and store genetic materials, particularly local species; to introduce genetic material stored in international institutions; to store plant gene pools material in Turkey for future use in plant improvements and species diversification studies. Modern biotechnological methods are known to be especially effective in the achievement of these goals.

Isogenic lines, nullisomic lines, substitution lines, addition lines, doubled haploid populations, mutants etc. revealed by biotechnological and classical genetic studies contribute to the increase of the diversity. Biotechnological applications also help for better understanding of the genetic structure of developed genotypes. This will be an important cornerstone for better recognition of the plant gene pools in Turkey and ensuring efficiency of the protection strategies aimed at local and original genes.

**Figure 1.**
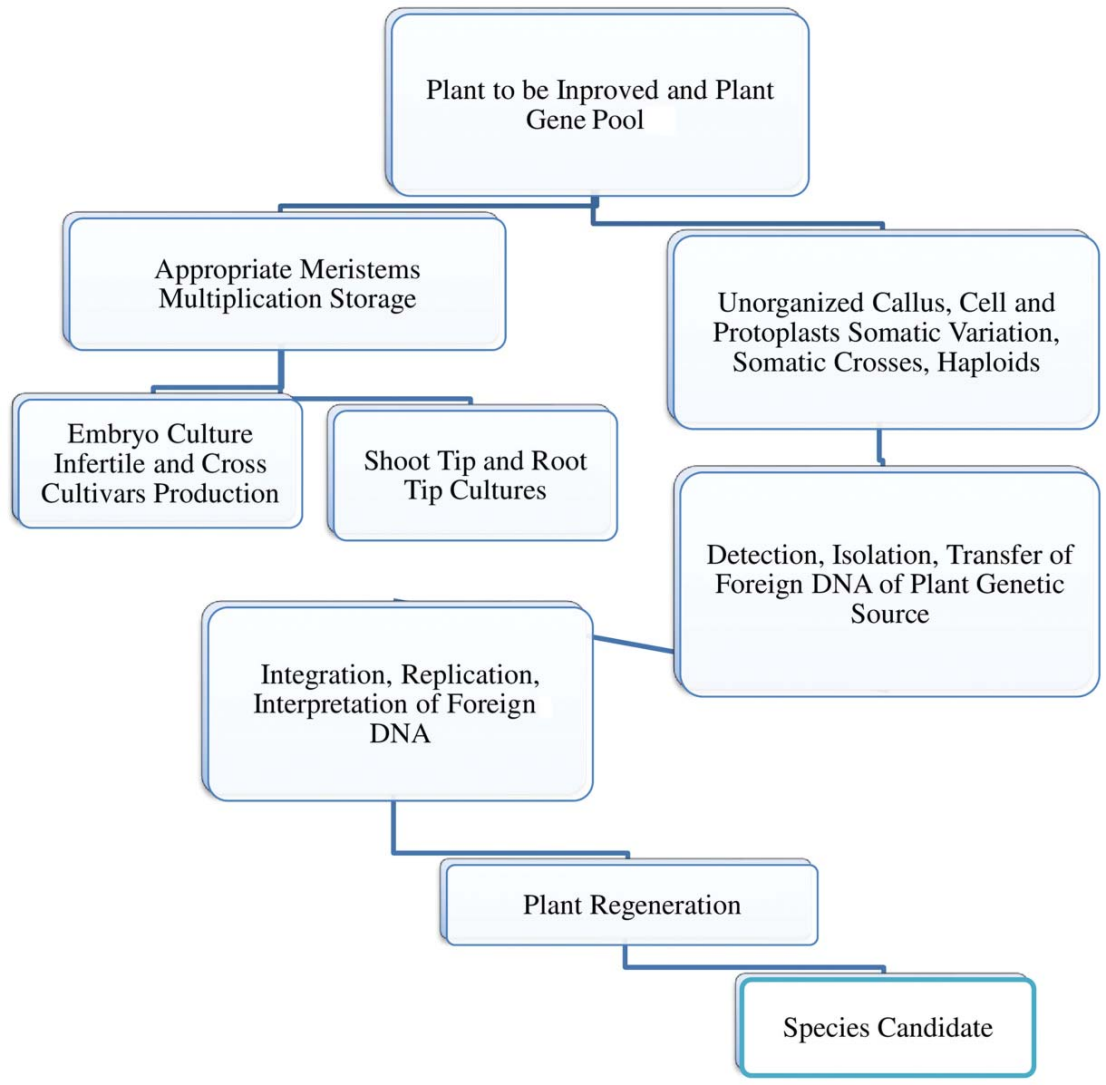
Role of biological gene pools in plant improvement studies using biotechnological methods.

Cross studies conducted using biotechnological methods also play a role in increasing the diversity. At the same time, the use of biotechnological methods can circumvent problems encountered in classical crossbreeding studies, such as infertility, incompatibility and linkage. Because *in vitro* selection enables selection of cells rather than of whole plants, this means working on Petri dishes at the cellular level rather than on thousands of plants in the field. Another important advantage is that there is no need to rely on developmental stages of plants for selection, since selection can be made at any time in *in vitro* conditions (Figure 1).[3]

Moreover, marker-assisted selection studies create opportunities for transfer of target genes from wild relatives to cultured species. Since the real distance between the molecular markers on chromosomes can be calculated, in plant species these maps can be widely used in gene mapping studies based on recombination frequencies. In addition, with the help of these markers, a long distance has been covered in the mapping and revealing of quantitative trait loci effects which are related to characteristics such as yield, quality, plant height and flowering time and which are controlled by many genes and are affected by environmental conditions. Besides, biotechnological methods make significant contributions to the development and spread of plants which are resistant to environmental conditions such as high or low temperatures, salinity, drought or excess moisture, or have an extended shelf life.

Another field where biotechnological methods are adopted in Turkey is landscaping: plant diversity is created in the field of landscaping by adding new traits to various plants used in landscaping. Clove is a good example for extension of shelf life. While natural cloves can survive in water up to 10 days, cloves subjected to biotechnological applications can survive up to 22 days without losing any trait. This situation increases the value of clove in its use as an ornamental plant in home, garden and landscaping applications, and the ability to stay alive for quite a long time makes clove more preferable over other delicate ornamental plants.[3] One of the most colourful fields of application of biotechnological methods is the creation of flower colours which are not encountered in nature under normal conditions. The flower colours of many plants such as rose, orchid and clove can be changed by this way. Blue cloves, which are not normally blue in colour, serve as a good example. However, scientists have successfully isolated the blue colour gene from petunia and transferred this gene to cloves, and many other flowers such as African daisy and chrysanthemum (for review see [18]). These are patented products currently commercially available in the market.

Owing to all of its aspects listed above, biotechnology plays a leading role in the protection of plant genetic pools and in taking and implementation of decisions on the sustainable use of these pools. Biotechnology is an indispensable tool particularly for sustainable agriculture. However, it should not be ignored that along with being a cornerstone in these dramatic developments, biotechnology bears some risks and the precautionary principle should be pursued.

## Possible negative effects of biotechnology applications on genetic diversity and interspecies gene exchange

The evolution of living organisms is a natural process ongoing for millions of years. During the whole evolution process, micro mutations in species may lead to larger, although quite rare, natural mutations. Among all genotypes resulting from these mutations, the ones that are able to adapt to changing environmental and stress conditions can continue their existence. Along with these changes happening throughout generations, phenotypic changes also happen for better adaptation to environmental and stress conditions. For instance, among different species of the same genus, the ones growing in cold regions are relatively shorter and have wider surfaces. Similarly, during their natural evolution process in regions with high pest populations, the plants develop natural defence mechanisms such as thickening of the cell wall, mechanical defences, e.g. prickles, spines, thorns, trichomes (hairs), raphides, etc., forming a waxy layer on the outer surface. Meanwhile, genotypes resistant to diseases evolve as well. However, pests, in turn, also evolve naturally to develop natural resistance mechanisms against these defences. Pathogens also evolve to circumvent the effect of the resistance genes that plants have developed. For this reason, some cultured species recorded to have resistance against a specific disease are sometimes struck by newly developed strains of the same pathogenic agent in a period as short as a few years.

Genes are exchanged between species in nature and there is an abundance of crossbred plants and even new species that have resulted from gene exchange between species in nature. This issue becomes important in terms of activation of resistance genes acquired from natural resistance mechanisms in the evolutionary process. What turn the evolutionary process of natural flora will take via the transgenes received from outside, is an open question.

As a result of the biotechnological advances in recent years, gene exchange between different life forms is now also possible. In line with these developments, discussion has been provoked on the potential threat of genetically modified organisms (GMOs), which are claimed to be developed to meet the food demand of the rapidly increasing world population but also to be able to negatively affect biological diversity in the long term. These discussions are focused on the possibility for the natural evolution process to change in an undesired way by the genes introduced unnaturally and the important threat such a situation may pose on gene pools. There may even arise problems which are more difficult to overcome than those existing now.

Gene flow is possible from genetically modified cultured species to their wild relatives. Thus, evolution ongoing for millions of years may change direction in a short period of time, like 40–50 years, as a result of contamination of natural plants by genes from GMOs. These are rates of change with which biological diversity cannot keep apace and thus, GMOs pose the risk of changing the course of the evolutionary process in an undesired direction. That is why GMOs constitute a potential threat to biological diversity and sustainable agriculture. This is particularly important for the rare and endemic species for which Turkey is the diversity and gene centre, as well as for crop species.

### Crop species

Crop plants are especially vulnerable to some of the possible risks posed by biotechnology applications. The total GMO cultivation area in the world in 2013 covered 175 million hectares.[19] These products and their cultivation areas are listed as 100 million hectares of soybean, 160 million hectares of corn, 33 million hectares of cotton and 31 million hectares of canola. Among these plants, 21% of the total cultivation area of canola is allocated for GMO production.[2,19,20] Thus, enlarging of GMO cultivation areas turns out to be a factor increasing the possibility of gene transfer between GMO products and their wild relative species. Cultivation areas are small and fragmental, particularly in countries with rich plant diversity, such as Turkey, which constitutes an important problem in view of GMO's. Higher use of GMO products in cultivation areas will endanger the original species due to gene flow and gene spread/transfer.

Resistance genes introduced into cultured GMO species have some characteristics different from those of the ordinary resistance mechanisms. For instance, bacterial Bt genes transferred to corn, cotton and potato for protection against pests result in sudden death of plant-feeding pests due to the toxins they produce. In case of heavy use of these species on large cultivation areas, the number of natural enemies of the target pests will start to decrease in parallel with the decrease in the pest population. Besides, as a result of the transfer of some insect-resistance genes to crop plants, a group of insects, including beneficial ones, would be destroyed indiscriminately. On the other hand, there is real possibility for these genes to be transferred into wild species through natural crossbreeding and, thus, the wild species to develop resistance against pests.

For instance, laboratory studies [21] have been conducted on genetically modified corn pollens and monarch butterfly larvae (*Danaus plexippus*) exposed to toxic amounts of Bt pollen on their host plant, the common milkweed, *Asclepias syriaca*, found in and around cornfields. The results showed lower growth rate and higher death frequency, depending on the vulnerability of the larvae, even at quite low toxin concentrations as compared to larvae feeding on normal pollens.[21] Although the effects may be interpreted as negligible, these observations underline the importance of the issue. What is more, Bt toxin enters into the nutrient cycle of living things via contamination of soil and water by the leaves of transgenic plants.

By analogy, due to possible gene flow from herbicide-resistant cultured species to their wild relatives, the latter may develop resistance against herbicides as well. Thus, serious problems would be faced if some weed and pest species which we can now fight by using existing technologies and chemicals develop into new uncontrollable resistant forms which may, consequently, do more harm to plants. In such case, the solution would be to develop and use chemicals that are much more effective (and/or more concentrated) than the ones presently existing. Paradoxically, the very technologies intended to reduce chemical use would actually result in more severe environmental damage due to the need to use more toxic chemicals which may in some cases prove irremediable.

### Poaceae

In terms of crossbreeding between species, there are some notable families and plant groups in Turkey. Among these, wheat from the Poaceae family has an evolutionary process full of examples of interspecies gene exchange. As it is known, all wheat species cultured today are artificial species formed as a result of crossbreeding between their wild relatives. The existence of crossbred species developed by pollination of wheat species (which have developed as a result of past interspecies genome exchange) by other species can be accepted as a part of natural evolution. This is an illustration of the possibility for these species to receive genes from transgenic plants. Studies point to gene flow between bread wheat and its wild relative *Aegilops cylindrica.*[17,22] In addition, a high number of studies show that there are natural and artificial crossbreeds between *Agropyron*, *Elymus*, *Festuca*, *Lolium*, *Hordeum*, *Triticum* and species of many wheat genera which naturally grow in Turkey. Among these studies, Belanger et al. [23] reported crossbreeding among bent grass (*Agrostis*) species; Ellstrand [24] reported 0.5% crossbreeding among setaria (*Setaria*) species and 39% breeding among pennisetum (*Pennisetum*) species and that this ratio increased up to 100% among *Sorghum bicolor* and *Sorghum halepense*. In addition, according to Fedak [25], barley (*Hordeum vulgare*) and intermediate wheatgrass (*Agropyron intermedium*) can develop crossbred plants with 3.9% crossbreeding. Of special interest is a possible gene flow from soybean, corn, cotton and rapeseed – with a total cultivation area of 7.2 million hectares in the world (as of 2013) – to their wild relatives.

### Brassicaceae

Another family at risk in terms of interspecies crossbreeding in Turkey is the mustard family (Brassicaceae). Many species of this family are known to be used in different ways.[26] For instance, their tubers, stems, leaves, flowers and seeds are consumed as food; some species (e.g. *Alyssum saxatile*, *Brassica oleracea*, *Cardaria draba*, *Crambe orientalis*, *Iberis saxatilis*, *Isatis glauca*, *Lobularia maritima*, *Matthiola incana*) are used in landscaping as ornamental and cover plants; while others (e.g. *Capsella bursa-pastoris*) are used in the pharmaceutical industry as medical plants or as dye-bearing plants (e.g. *Isatis tinctoria*) in the textile industry.[27] There are many reports suggesting an extensive gene exchange between the species of the mustard family. According to 2013 data, the global GMO-containing rape production is 21%.[19] The gene used for genetic modification in rape is a herbicide resistant gene. Rape is an interspecies hybrid of two different species (*Brassica napus* × *Brassica campestris*) rather than being an original species. Elsstrand [24] suggested that the possibility for gene flow from *Raphanus sativus* to its namesake wild relative is 100%; Ford et al. [26] pointed out a similar risk. In the People's Republic of China, genes of genetically modified, herbicide-resistant rape (*Brassica napus*) have been detected to have escaped to mustard greens (*Brassica juncea*), the free-living wild relative.[19]

### Amaranthaceae

The Amaranth family (Amaranthaceae) is also known to be one of the families with high gene flow. Desplanque et al. [28] pointed out the possibility and probability of gene flow from sugar beet to wild goosefoot and the negative impacts of the transgenes escaping from herbicide-resistant sugar beet to nature.

### Biofuel species

Another topic of discussion on the potential negative effects of GMO implementations are the species used for biofuel production. In particular, the European Union (EU) countries have set future targets for use of biofuels produced from materials such as plant origin oils, product residues and wood. The EU targets to meet 20% of its fuel demand from biofuels by the end of 2020 and supports farm production of rape for biofuel.[22] The cause of concern, particularly with enlargement of GMO rape cultivation areas to achieve the ‘20% target’ in biofuel use, stems from the risk of gene flow from rape to wild relative forms and cultured mustard species (see above). This would obviously result in damage to agricultural sustainability and agricultural gene pools.

All these examples should be given careful consideration, as indiscriminate use of GMOs can result in disruption of ecological balance and, finally, in damage to plant gene pools. For this reason, the possible negative impacts on plant gene pools should be monitored closely and plant origin genes should be given priority in this type of studies.[3]

## Biotechnological research and legislation in Turkey

In the context of environmental protection, it can be suggested that molecular plant improvement methods can be more advantageous than transgenic plants in developing counties such as Turkey. At this point, detection and characterization of Turkish genetic pools and even defining and patenting those of commercial importance will elevate Turkey to a high position. Once the International Treaty on Plant Genetic Resources adopted by FAO in 2001 is enacted [11] and Turkey establishes the required legal and research infrastructure, there will be more space to utilize genetic pools more efficiently. However, examination of the current situation in Turkey shows that biotechnology implementations cannot be transferred to industry and the required infrastructure has not been established yet. Institutional structuring of the countries with highly developed biotechnology is based on three pillars: universities, public research and development (R&D) organizations and the private sector. Each pillar has its own roles in line with its capacity and work definition. For instance, while universities and public R&D organizations are specialized in basic research, the private sector concentrates on applied research and product development. Failure of an organization which is within the system and serves as a complement to others, results in dysfunctioning of the whole system. From this point of view, a brief overview of the current structure in Turkey shows that universities serve as the basic component of the research system and the private sector is not included in the system but indeed should be a main component. Therefore, since there is no institutionalization to regulate the roles of the private sector and public R&D organizations, there is no target-oriented system operating efficiently.[29] This systemic gap prevents the biotechnological solutions successfully developed in university research laboratories to be applied in the field. Due to the above-listed problems, Turkey does not have the infrastructure required for cultivation of GMOs despite its strong position on this issue. Therefore, it is now not allowed to grow GMOs in Turkey. Thus, there is no commercial production of genetically modified crops in Turkey and Turkey does not export such crops to the United States or other countries. In 2013, Turkey continued to be an importer of bulk and semi-processed commodities. Cotton remained the top exported commodity from the United States to Turkey in 2013. Soybeans and soybean meal are the second largest imported commodity, but their portion has decreased dramatically since 2011 and 2012.[30]

There is no ban, however, on production of GMOs for research and development purposes in Turkey. In addition, import and production of 19 types of products (3 soybean and 16 corn species) are permitted only for animal feeding purposes.[10,31] These fodders have all or some of the characteristics of resistance to corn pests and tolerance to the herbicides glyphosate and glufosinate ammonium.[31] Import of these products in Turkey, although subject to some limitations, seems to clear the way for cultivation of GMO products in the near future. Attempts made in the last couple of years to consolidate small cultivation lands in Turkey support this suggestion. Detection of the potential risk faced by plant gene pools, performance of the infrastructure works to minimize this risk and taking all required precautions are the three most important points to be underlined here. For maximum utilization of the existing genetic potential, Turkey should develop sufficient infrastructure for biotechnological studies by defining the areas of priority and should train enough qualified specialists.

From a legislative point of view, Turkey introduced legal regulations on the production and inspection of GMO-containing products under the Law on Biosafety in 2010. Turkey's Law on Biosafety permits regulations on the use of GMOs and development of plant biotechnology. However, the cumulative disincentives in the forms of quarantine control, approvals, liability and prohibition on the cultivation of biotechnological products have discouraged the development of GMOs. The law also mandates that for any research on GMO development carried out in Turkey, the researcher needs to apply to the Biosafety Board in advance for permission to carry out the research. Although many academicians voiced concerns about this issue, and the Ministry of Food, Agriculture and Livestock (MinFAL) has stated that the law will not discourage research, it seems to have already had an impact on the willingness of the private and public sector to pursue research in this area.[30] Turkish companies and universities have so far not developed any transgenic seeds. Turkey does not have any field testing of products derived from agricultural biotechnology.[30] However, this law allows for import and production of transgenic soybean and corn that can be used only as fodder. The law forbids entrance into Turkey of GMOs and GMO-containing products, which threaten human, animal and plant health as well as environmental and biological diversity; disrupt the ecological balance of the environment and ecosystems; carry the risk of spreading in the environment and threaten the continuity of biological diversity. Issues such as GMO content, inspection, import, labelling, etc. of GMO-containing products are regulated by the Biosafety Commission established by this law and the MinFAL of Turkey. In this context, Turkey can be suggested to pursue a prudent policy on protection of national genetic pools, although perhaps not at a sufficient level.

## Conclusions

Throughout its historical development, biotechnology has played significant roles in the protection, use and diversification of all components of biological diversity. It has been a cornerstone particularly in the protection of plant gene pools and in ensuring agricultural sustainability. Biotechnological methods continue to be used in the protection of plant gene pools, which are difficult or impossible to protect via classical methods. In its own right, biotechnology is an indispensable means not only for sustainable management of plant gene pools, which includes the insurance of sustainable agriculture and sustainable use of gene pools, but also for creation of new diversity resources.

However, the use of biotechnology for development of Bt-toxin-producing plants may damage the ecological balance and plant gene pools as a result of contamination of nature with undesired genes. There are many examples of interspecies gene exchange during evolution in nature. A careful observation of nature suggests that interspecies gene flow is an ongoing process and, in turn, it is possible for gene flow to occur from GMOs to wild relatives. However, the results of such gene flows cannot be observed in the short term.

Thus, GMOs can potentially pose a significant threat on genetic diversity unless required measures are taken and possible risks are considered. This situation is particularly important for areas rich in populations of wild relatives of crop plants (e.g. Turkey, which is a genetic and origin centre for many crops) and for some plant families with high interspecies crossbreeding. Having rich biological diversity, Turkey should take necessary precautions to protect its plant gene pools and ought to pursue policies aimed at sustainable development by taking appropriate protection measures. Turkey can be a power focus with its rich plant gene pool diversity through protecting its original and local species only if it gives particular priority to the application of biotechnology and duly pursues biotechnology policies that are in line with the precautionary principle.
